# Nasal Hemorrhage

**Published:** 1850-01

**Authors:** 


*Nasal Hemorrhage.*—There are few physicians who have not occasionally been annoyed by the difficulty with which nasal hemorrhage is arrested. An old ship master communicated to me a method, which shows that the artery furnishing the supply of blood can be perfectly compressed at the root of the upper incisor teeth. His process was to roll up a piece of paper and place it under the upper lip. The first opportunity I had of trying it, was a case of profuse hemorrhage from a fall, which had persisted four days, notwithstanding repeated plugging of the nostrils, and the patient had become almost exsanguine. In this case the front teeth of the patient were wanting, and I applied the pressure by tying a knot in a bandage, which I placed *on* the upper lip so as to make pressure immediateiy at the root of the septum narium, and tied the bandage around the head above the ears. The hemorrhage was immediately and permanently arrested. On mentioning the subject to several of my medical friends, I found the practice was new to them all, and I therefore communicate it for the benefit of the profession.

SAMUEL R. SMITH.

*Tompkinsville, Staten Island, N. Y., Oct.* 15, 1849.

*Boston Med. and Surg. Journal.*

